# Two Different Cell Populations Is an Important Clue for Diagnosis of Primary Cutaneous Adenoid Cystic Carcinoma: Immunohistochemical Study

**DOI:** 10.1155/2017/7949361

**Published:** 2017-01-24

**Authors:** Banu Ince Alkan, Onder Bozdogan, Müjde Karadeniz, Nazan Bozdoğan

**Affiliations:** ^1^Pathology Department, Abdurrahman Yurtaslan Oncology Education and Research Hospital, Ankara, Turkey; ^2^Department of Pathology, Numune Education and Research Hospital, Ankara, Turkey

## Abstract

Primary cutaneous adenoid cystic carcinoma (PCACC) is a very rare malignancy. The differential diagnosis of PCACCs in pathology practice can be difficult and a group of primary and metastatic lesions, including adenoid basal cell carcinoma of the skin, should be considered in the differential diagnosis. Besides histomorphological clues, immunohistochemistry studies are very helpful in the differential diagnosis of PCACC. We report herein a case of PCACC with extensive immunohistochemical studies and review the literature from an immunohistochemistry perspective.

## 1. Introduction

Primary cutaneous adenoid cystic carcinoma (PCACC) is a very rare malignancy of the skin [1]. The histopathology of PCACC is very similar to adenoid cystic carcinoma (ACC) at other sites [2]. PCACCs have a tendency to recur locally but rarely show metastasis to the lymph nodes or distant organs [3]. We report herein a case of PCACC with immunohistochemical (IHC) findings and review the literature from an IHC perspective.

## 2. Case Report

**Figure 1:**
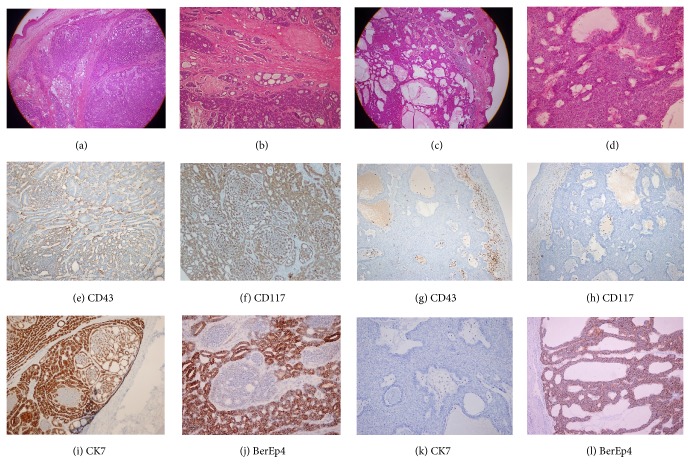
Primer cutaneous adenoid cystic carcinoma (PCACC) (left two columns). Adenoid basal cell carcinoma (A-BCC) (right two columns). (a-b). Typical regular cribriform pattern is readily detected in PCACC. (c-d). However, pseudoluminal areas are not regular, and the well-defined cribriform patterns are not seen in A-BCC. CD117 (f) and CK7 (i) are positive in PCACCs but not in A-BCC (h, k). CD43 positivity is heterogenoeus in PCACC (e) but no positivity is detected except for inflammatory cells in A-BCC (g). BerEp4 positivity is seen in both lesions (j, l). A-BCC shows diffuse positivity (l), but only ductal cell positivity in PCACC (j). Original magnifications: (a, c) ×40; (b, e, f, g, h, i, k, l) ×100; (d, j) ×200.

A 54-year-old male patient presented with a nodular lesion on his back. Physical examination showed a nodular, oval cutaneous lesion without specific features. The lesion was a grossly well-circumscribed, gray-tan, 2 × 2 × 1.5 cm intradermal nodule. Microscopic sections revealed tumor cells, which were arranged in nests, tubular patterns, cribriform patterns, and solid islands (Figures 1(a) and 1(b)). The cell nests consisted of two cell types: polygonal cells, which were localized at the center of the islands or around luminal and cystic spaces, and basaloid cells, which were located at the periphery of the islands. Solid islands were also formed from basaloid cells. The basophilic/eosinophilic granular secretions were readily detected in the lumina. The lesion had no connection with the epidermis and no lymphovascular space or perineural invasion. Mitoses were readily detected. To compare the histopathology and immunohistochemistry of the tumor, a case of classical adenoid-type basal cell carcinoma (A-BCC) in an 88-year-old female was also studied (Figures 1(c) and 1(d)).

An IHC study was performed for two patients using the Ventana Benchmark Ultra automated immunohistochemistry system (Ventana Medical Systems, Inc., Tucson, AZ, USA). CK7 (1/200, monoclonal; Thermo Fisher Scientific, Inc., Waltham, MA, USA), CEA (1/200, monoclonal; Thermo Fisher Scientific, Inc.), EMA (1/200, monoclonal; Cell Marque; Sigma-Aldrich, St. Louis, MO, USA), BerEP4 (1/50, monoclonal; Cell Marque), Laminin B2 (1/100, monoclonal; Thermo Fisher Scientific, Inc.), CD117 (1/200, polyclonal; Thermo Fisher Scientific, Inc.), and CD43 (1/100, monoclonal; Ventana) were applied to paraffin sections of both cases. Further IHC staining examinations were performed on paraffin sections of the PCACC case; these examinations included S100 (1/200, monoclonal; Cell Marque), collagen 4 (1/50, cocktail; Thermo Fisher Scientific, Inc.), p63 (1/100, monoclonal; Biocare Medical, Concord, CA 94520, USA), SMA (1/200, monoclonal; Thermo Fisher Scientific, Inc.), and CK5/6 (1/100, monoclonal; Thermo Fisher Scientific, Inc.).

**Figure 2:**
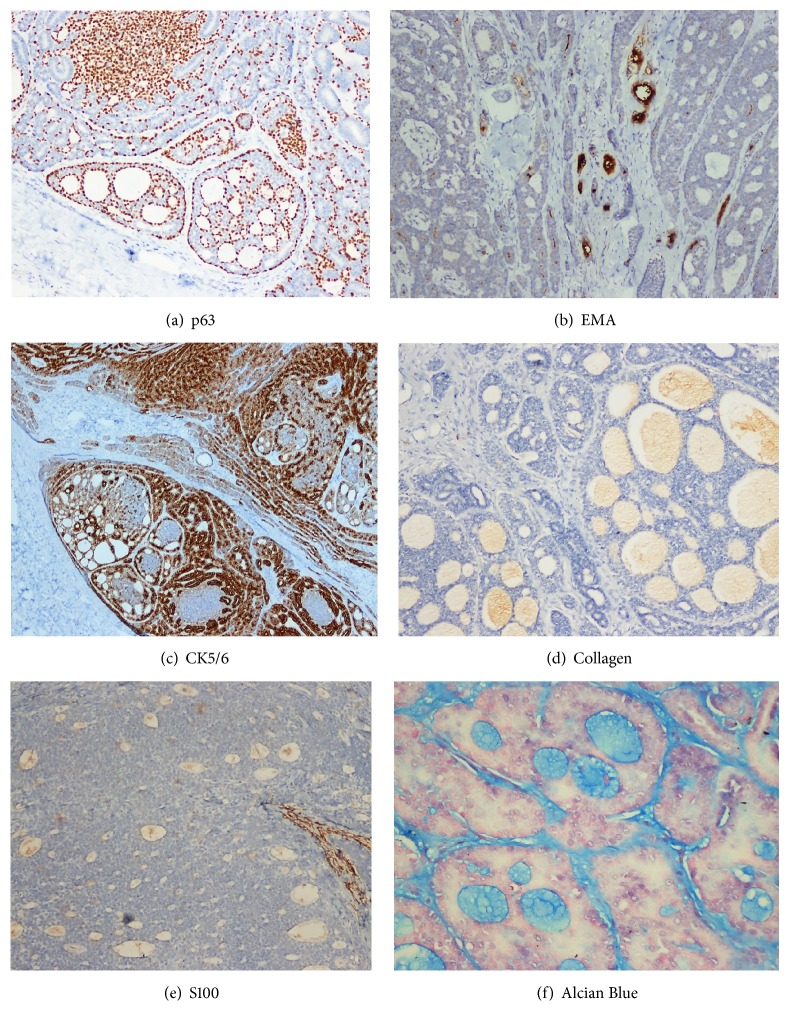
Primary cutaneous adenoid cystic carcinoma. (a). p63 highlights the myoepithelial cells. (b). EMA shows positivity in only a few lumina. (c). CK5/6 positivity in PCACC. (d). Although collagen 4 positivity has weak or medium intensity, it highlights the luminal material. (e). S100 is negative in our case and only positive in dendritic cells. (f). Alcian Blue positivity in luminal secretion. Original magnification: (a, b, c, e) ×100; (d) ×200; (f) ×400.

In the lesion, the two different areas (luminal and basal) were differentiated according to the IHC results, which is similar to the histology. The polygonal (ductal/epithelial) cells were strongly stained by CD117, CK7, and BerEP4 (Figures 1(f), 1(i), and 1(j)). The basaloid (myoepithelial) cells showed positivity for SMA and p63 (Figure 2(a)). CEA and EMA positivity were also detected in the small number of luminal areas (Figure 2(b)). Laminin B2 and CD43 showed heterogeneous and medium intensity positivity (Figure 1(e)). CK5/6 was also strongly positive (Figure 2(c)). Collagen 4 positivity highlighted the luminal material (Figure 2(d)). However, S100 positivity was not detected (Figure 2(e)). The A-BCC case showed no real glandular lumina on histopathological examination, and there was no clear-cut differentiation between the two cells. The A-BCC case showed diffuse BerEP4 positivity but was negative for EMA, CK7, CD117, CEA, Laminin B2, and CD43 (Figures 1(g), 1(h), 1(k), and 1(l)).

Clinically, to exclude the possibility of a metastatic lesion, the patient underwent further radiological examinations. However, no further lesion was detected. Therefore, the case was diagnosed as primary cutaneous adenoid cystic carcinoma (PCACC) grade 2 using the clinical, morphological, and IHC data [27].

## 3. Discussion

Although ADCCs of the salivary gland and the upper airways are commonly observed in routine clinical practice, PCACCs are very rare tumors, with <100 cases, including small series, having been reported previously [1, 26, 28].

Histopathologically, PCACCs consist of basaloid cells, which are arranged as cribriform nests, tubules, cords, and solid areas in the dermis and subcutis [1, 29]. The luminal areas usually consist of alternating eosinophilic or basophilic secretions [1]. The tumor cells show two distinct types of differentiation: ductal/epithelial differentiation around pseudocysts and myoepithelial differentiation in the outer layers of cell nodules [2, 16].

The differential diagnosis of PCACCs in pathology practice can be difficult. Adenoid basal cell carcinoma, primary mucinous carcinoma of the skin, metastatic breast carcinoma, eccrine adenoma, syringoma, mixed tumor of the skin, metastasis from primary ACCs of the salivary gland, and rare primary cutaneous cribriform carcinoma should be considered in the differential diagnosis [1, 25, 30]. The importance of immunohistochemistry has been well demonstrated in the differential diagnosis of PCACC [13]. Because the tumor cells show ductal and myoepithelial differentiation, IHC markers highlight different positivity in two different cell populations. In the present case study, PCACC showed BerEp4, CEA, CD117, and CK7 expression in the regions neighboring the luminal areas. p63 and SMA positivity were detected in myoepithelial cells at the periphery of the cell islands. The two different cell populations provide an important clue for diagnosis.

Due to the problems of differential diagnosis, groups of IHC markers were examined in the previous literature (Table 1), and the main differential diagnosis was between PCACC and A-BCC. Although peripheral palisading of tumor cells, continuity with the epidermis or adjacent hair follicle, and retraction artifacts between the tumor island and stroma are important features of A-BCC, distinction may not be easy in routine pathological investigations especially small biopsy specimens (Table 2) [31]. Therefore, immunohistochemistry may be helpful for differential diagnosis in the presence of these two similar entities [13]. Dessauvagie and Wood emphasized the importance of the CD117 and CD43 antibodies. CD117 positivity was present in all of the ACCs, and CD43 positivity was present in 40% of the ACCs. The BCCs showed no CD43 staining, and only 20% of the A-BCCs were positive for CD117 [13]. We also found CD117 and CD43 positivity in the PCACC but not in the A-BCC. However, a conflicting report was published by Terada, who showed that 93% of BCCs were positive for CD117 [32]. Our experiences showed that CK7 positivity is rare in BCC and assists in the differentiation between sweat gland lesions and BCCs. The classical markers, CEA and EMA, are expected to be positive in the ductal cells of PCACC but generally not in classical A-BCC [1, 31]. Furthermore, the two different cell populations are not detected in A-BCCs.

The cribriform areas of spiradenomas should also be considered in the differential diagnosis. However, this change usually occurs focally, and the typical morphological features of spiradenomas are readily detected. Although the two cell populations are detected in spiradenomas, this pattern is less organized than that in PCACCs (Table 2) [33]. Although primary cutaneous cribriform carcinomas (PCCCs) are so rare, they should also be considered in the differential diagnosis. PCCCs are well circumscribed showing epithelial attenuation at cystic spaces and intraluminal micropapillae. However, they may express CD117 positivity [25, 30]. The two cell populations, which can be highlighted using myoepithelial markers, are not found in PCCCs (Table 2) [30].

Histomorphologically, unlike the ACC of salivary glands, PCACCs show nodular growth patterns and perineural invasion only observed in 50% of the cases [1]. However, due to similar morphology and IHC staining patterns, the differential diagnosis of PCACC and metastatic ACC can only be distinguished based on clinical grounds [31].

In conclusion, the differential diagnosis of PCACCs can create difficulty. In addition to clinical and morphologic data, IHC evaluation may be helpful when used with a suitable panel of markers that highlight the two cell populations.

## Figures and Tables

**Table 1:** Summary of immunohistochemical studies in the literature^&^.

IHC marker	Positivity	Percentage	Notes	References
PAN-keratin	10/10	100%	Including AE1/AE3 keratin	[4–11]
Alfa-lactalbumin	1/1	100%		[9]
Amylase	1/1	100%		[9]
Ber-ep4^*∗*^	1/2	50%		[12]
Blood group isoantigens	0/1	0%		[9]
B2-microglobulin	0/1	0%		[9]
Calponin	2/2	100%		[11]
CAM5.2	0/1	0%		[5]
CD10	1/2	50%		[5, 12]
CD43^*∗*^	2/3	66,6%		[13]
CD56	1/1	100%		[5]
CD57 (Leu7)	0/1	0%		[8]
CD117^*∗*^	37/37	100%		[1, 5, 6, 12–16]
CEA^*∗*^	24/33	72,7%	Focal, luminal positivity	[1, 5–8, 11, 12, 17–22]
CK5/6^*∗*^	17/17	100%		[5, 14]
CK7^*∗*^	20/20	100%		[5, 10, 12, 14]
CK10	0/1	0%		[10]
CK15	13/14	92,8%		[14]
CK18	1/1	100%		[10]
CK19	1/2	50%		[5, 12]
CK20	0/1	0%		[5]
D2-40	13/15	86,6%		[14]
EMA^*∗*^	27/30	90%	Focal, luminal positivity	[1, 4–6, 8, 9, 11, 12, 17–19, 22, 23]
GCDFP-15	0/1	50%		[12]
HMWCK	6/6	100%	Including 34Be12 clone	[5, 11, 17, 18, 21]
Laminin^*∗*^	3/3	100%		[17, 24]
LMWK	4/4	100%		[8, 17, 18, 21]
MNF-116	14/14	100%		[1, 22]
P16	1/1	100%		[16]
P63^*∗*^	17/19	89,4%		[5, 12, 14]
Peanut agglutinin (PNA)	1/1	100%		[9]
S-100^*∗*^	28/30	93,3%	Generally focal positivity	[1, 5–9, 11, 12, 17–23]
SMA^*∗*^	33/36	91,6%	Including one immunoflourescent study	[1, 5, 10, 11, 14, 18, 20]
SOX-10	19/19	100%		[14]
Type IV collagen^*∗*^	12/13	92,3%		[1, 17, 24]
Vimentin	13/16	81,2%		[8, 10, 14]

[4–12, 14, 15, 17–24].

^&^Adenoid cystic carcinoma of the eye and eyelid excluded. ^*∗*^Including this case.

**Table 2:** Differential diagnosis of primary cutaneous adenoid cystic carcinoma.^*∗*^

Main differential diagnosis	Morphologic clues	Immunohistochemistry	Other
Adenoid basal cell carcinoma	(i) Peripheral palisading (ii) Retraction artifact (iii) Continuing with epidermis or hair follicle (iv) Lack of two cell populations	(i) CEA, EMA negative (ii) CD117, CK7 usually negative	Although the staining pattern is not the same, Ber-Ep4 may not be very helpful, with positivity in both lesions.

Primary cutaneous cribriform carcinoma	(i) Epithelial attenuation (ii) No perineural invasion (iii) Micropapilla formation (iv) Lack of two cell populations	(i) Myoepithelial markers (p63, calponin, and SMA) usually negative	CD117 is not helpful, with positivity in both lesions.

Metastatic ACC	(i) Similar morphology	(i) Similar immunohistochemical findings	Differential diagnosis should be done on clinical grounds.

Cribriform patterns in spiradenomas	(i) Focal cribriform areas with typical spiradenoma morphology (ii) Two cell populations may be found but may be more irregular	N/A	ACC-like areas show myoepithelial differentiation and may be positive with p63 and SMA.

Metastatic breast carcinoma	(i) Lack of two cell populations (ii) Cancer cells in lymphovascular spaces	(i) Myoepithelial markers (p63, calponin, and SMA) usually negative	Strong ER and PR may point out metastatic breast carcinoma, but adnexal neoplasms may also be positive.

^*∗*^This table is established by using [1–3, 13, 16, 25–33]. ACC: adenoid cystic carcinoma.
